# Assessing Mammal Exposure to Climate Change in the Brazilian Amazon

**DOI:** 10.1371/journal.pone.0165073

**Published:** 2016-11-09

**Authors:** Bruno R. Ribeiro, Lilian P. Sales, Paulo De Marco, Rafael Loyola

**Affiliations:** 1 Laboratório de Biogeografia da Conservação, Departamento de Ecologia, Universidade Federal de Goiás, Goiânia, Goiás, Brazil; 2 Programa de Pós-graduação em Ecologia e Evolução, Universidade Federal de Goiás, Goiânia, Goiás, Brazil; 3 Laboratório de Metacomunidades e Ecologia de Paisagens, Departamento de Ecologia, Universidade Federal de Goiás, Goiânia, Goiás, Brazil; 4 Brazilian Research Network on Climate Change–Rede Clima. Instituto Nacional de Pesquisas Espaciais, São José dos Campos, São Paulo, Brazil; Sichuan University, CHINA

## Abstract

Human-induced climate change is considered a conspicuous threat to biodiversity in the 21^st^ century. Species’ response to climate change depends on their exposition, sensitivity and ability to adapt to novel climates. Exposure to climate change is however uneven within species’ range, so that some populations may be more at risk than others. Identifying the regions most exposed to climate change is therefore a first and pivotal step on determining species’ vulnerability across their geographic ranges. Here, we aimed at quantifying mammal local exposure to climate change across species’ ranges. We identified areas in the Brazilian Amazon where mammals will be critically exposed to non-analogue climates in the future with different variables predicted by 15 global circulation climate forecasts. We also built a null model to assess the effectiveness of the Amazon protected areas in buffering the effects of climate change on mammals, using an innovative and more realistic approach. We found that 85% of species are likely to be exposed to non-analogue climatic conditions in more than 80% of their ranges by 2070. That percentage is even higher for endemic mammals; almost all endemic species are predicted to be exposed in more than 80% of their range. Exposure patterns also varied with different climatic variables and seem to be geographically structured. Western and northern Amazon species are more likely to experience temperature anomalies while northeastern species will be more affected by rainfall abnormality. We also observed an increase in the number of critically-exposed species from 2050 to 2070. Overall, our results indicate that mammals might face high exposure to climate change and that protected areas will probably not be efficient enough to avert those impacts.

## Introduction

Human-induced climate change increases species extinction risk and is considered a prominent threat to biodiversity during and beyond the 21^st^ century [1–3]. Novel (non-analogue) climate conditions are expected in almost a third (12–39%) of the global surface. Likewise, 10–48% of Earth’s extent is projected to experience disappearing of current climate conditions [4]. As climate changes, many species might be exposed to climatic conditions likely to exceed their physiological tolerance. That exposure will probably result in physiological stress [5], reduction in fitness [6–8] or even extinction [9], especially if species are unable to track other suitable environmental conditions or undergo some kind of *in situ* adaptation [10–13].

Tropical species are particularly at risk from climate change [14–16]. Despite the smaller amount of change expected in the tropics when compared to temperate regions, tropical species are currently living closer to their thermal safety margin [5,15–19]. Thus, even small changes in climate might have deleterious consequences on species long-term survival because high conservatism of upper thermal limits prevent species to develop adaptations to increased temperature that will probably exceed their thermal tolerance [20,21]. Further, tropical species are more likely to have to cope with climate change *in situ*, since shallower temperature gradients within tropical lowlands prevents species to disperse and track other suitable climatic conditions [14]. The combination of increase of non-analogous climate conditions, higher sensitivity, narrower climatic niches and geographic distributions, and higher species richness, set the tune for tropical species being more vulnerable to climate change [4,16,19,22].

Exposure to climate change varies geographically within species’ ranges because projected changes on temperature and precipitation are unevenly distributed around the globe (see Garcia et al. [23]). Therefore, estimating how much and where populations are most at risk is paramount to understand the effects of climate change on species. It is also critical to evaluate if these population in different parts of a species’ range are safe; and, therefore, we need to look at Protected Areas (PAs). PAs play a central role in averting negative consequences of climate change [24]. They are supposed to sustain different microclimate conditions in heterogeneous habitats that are essential for species to avoid extreme climatic conditions [21]. Additionally, PAs buffer species against other human-induced threats such as habitat loss and fragmentation.

The Brazilian Amazon is a perfect study case to understand species vulnerability to climate change for mainly two reasons. First, it is the largest intact tropical rainforest in the world covering around 5 million km^2^, which corresponds to 61% of the Brazilian territory. Second, the Amazon has one of the most vast species composition on Earth harboring one out every five plant species (40,000 vascular plants), several bird (1300) and mammal (399) species [25]. It holds the highest number of mammals species among all Brazilians biomes; from 701 Brazilian mammals, 399 occur the in Amazon [26]. Moreover, the conservation status of the Brazilian Amazon is particularly interesting. Although it has been transformed in a mosaic of altered environments due to population growth and economic activities [27], it also holds the largest network of protected areas existing in Brazil, covering at least 23.5% of its territory [25].

Here, we quantified mammal local exposure to climate change across species’ range and identify which areas in the Brazilian Amazon have the highest concentration of species with critical exposure to different climatic variables. We also assessed the effectiveness of Amazon network of PAs in buffering the impacts of climate change on species. To do so, we compared the number of critically-exposed species found in each PA to the expected values of species richness estimated by a null model that randomly allocated PAs within the Amazon while maintaining their size, shape and orientation. Our null models overcomes some previous shortcomings (see Lemes et. al. [28] and Ferro et al. [29]) by explicitly incorporating the current distribution of the network of Amazon PAs in randomization process, making the null model more realistic.

## Materials and Methods

### Species’ data

Data on species distribution was obtained from range maps for terrestrial mammals on the International Union for Conservation of Nature website (IUCN version 2015–2; www.iucnredlist.org/technical-documents/spatial-data). We followed Paglia et al. [26] to account for taxonomic differences and to check for species occurrences in the Amazon. A total of 376 mammal species inhabiting the Brazilian Amazon were analyzed in this work. Species were considered endemic to the Amazon when occurrences in Brazil were limited to that biome, according to Paglia et al. [26]. We overlapped each range map into an equal-area grid of 0.5 x 0.5 degrees of latitude/longitude (around 55 km at equator line; see comment on spatial resolution in the ‘Climatic data‘ section) containing 12,807 and 1,767 cells covering the full extent of species’ range and the extent of the Brazilian Amazon, respectively. We assumed that a species was present in a cell if any portion of its distribution map overlapped the respective cell.

### Climatic data

We selected four bioclimatic variables to quantify mammal exposure to climate change: mean annual temperature, temperature seasonality, maximum temperature of warmest month, and precipitation seasonality. Those variables encompass multiple climate dimensions representing general trends, variation (seasonality) and extremes (maximum) climatic relevant aspects that affect, direct or indirectly, species distribution and survival (see S1 Table for further details about climatic variable selection and collinearity among variables). Although other factors might also affect species distribution, several studies indicate that climate is the main driver of species distribution at large spatial scales [30–32].

We obtained bioclimatic variables relative to current (1950–2000) and future (2050 and 2070) periods from the WorldClim database (version 1.4; www.worldclim.org/version1). Current variables were generated from interpolation of observed data from weather stations [33]. We used future climate projections derived from 17 downscaled Atmosphere-Ocean General Circulation Models from CMIP5 (AOGCMs; ACCESS1-0, BCC-CSM1-1, CCSM4, CNRM-CM5, GFDL-CM3, GISS-E2-R, HadGEM2-AO, HadGEM2-CC, HadGEM2-ES, INMCM4, IPSL-CM5A-LR, MIROC-ESM-CHEM, MIROC-ESM, MIROC5, MPI-ESM-LR, MRI-CGCM3, NorESM1-M) for one high-emission greenhouse gases scenario (Representative Concentration Pathways, RCPs 8.5; IPCC 2013) to quantify species local exposure to climate change. Some climate models may have low accuracy for the Amazon. In order to address this issue, we removed the models (GFDL-CM3 and INMCM4) that exhibited the largest variation in relation to predicted average values of climate variables. By excluding models with extremely divergent predictions, we avoided substantial bias in the analysis. The most extreme greenhouse gas emission scenario RCP8.5 was chosen because it encompasses all set of greenhouse gases emission scenarios used in the IPCC’s fifth Assessment Report [34]. Also, the chosen scenario has predictions most likely to be supported in the future, because trends on greenhouse emission since 2000 have been closer to those predicted by RCP8.5 than any other hindcast [35,36].

Current and future bioclimatic variables were obtained at the resolution of 10 arc-minutes (~ 0.16° of latitude/longitude) and rescaled to our grid resolution using mean of values within 0.5° cell (see Hijmans et al. [33]). Weather stations are sparsely distributed on fairly populated regions, such as the Brazilian Amazon. The WorldClim climate data are therefore interpolated from probably insufficient records, which enhances uncertainty on expected values. Higher resolution climatic data (0.1° cell, for example) do not necessarily imply on a higher data quality and might be pure statistical artefacts. In addition, the 0.5° resolution was chosen to counterbalance the inaccuracies associated with applying high resolution climatic data to a relatively coarse IUCN species distribution maps [37]. Calculations were done in R version 3.2.2 [38] mainly using the *LetsR* [39] and *Raster* packages and their dependencies. All figures presented in this paper are original and were created in R and ArcGIS 10.1 (ESRI, Redlands, CA, USA).

### Estimates of species local exposure to climate change

To identify the most exposed cells to climate change across species geographic range, we followed three steps. First, we used current climate conditions (1950–2000) within the entire species’ range to identify the amplitude (maximum and minimum limits) of each climatic variable to which species are currently exposed within its range. This procedure is useful to identify areas within species’ ranges where future climate conditions will probably exceed current climatic variability. In addition, IUCN distribution maps are built upon minimum convex polygons of species’ occurrence records. Therefore, using the extreme climate values of environmental conditions associated with species’ records allows us to avoid uncertainties related to dataset source, because occurrence records and IUCN range maps would probably produce similar results.

Second, for each grid cell and each climatic variable we calculated the standard deviation of projections derived from the 15 AOGCMs. The mean of each cell-based projection minus their corresponding standard deviation was considered the value of each climatic variable in the future (2050 and 2070). In doing so, we incorporated the variation from different future climate projections by assuming the value that a particular climatic variable may achieve in future as the mean minus one standard deviation. Therefore, our approach is extreme because we selected the highest emission greenhouse gas scenario, but conservative for assuming the lower values of climate variables.

Third, we identified cells within species’ range in which future climate conditions may exceed the current climate conditions experienced by the species. Species for which future climate conditions exceed their current tolerances in more than 80% of their ranges were designated as “critically-exposed”. We chose this percentage because significant reductions (≥ 80%) of a species range is one criteria used by IUCN to qualify a species as critically endangered (IUCN 2016). Endemic species were also analyzed separately, to assess their exposure in relation to other groups. Finally, we mapped areas with highest concentration of critically-exposed species to each climatic variable separately and thereafter, we combine these maps to build a “combined” exposure map.

### Data on protected areas

We obtained federal and state spatial data on PAs from the Chico Mendes Institute for Biodiversity Conservation (ICMBio, www.icmbio.gov.br) and the Brazilian Ministry of Environment (MMA; www.mma.gov.br). We overlapped the polygons of PAs to our 0.5 x 0.5° grid. Not all overlapping cells were considered “protected”; only those for which PAs polygons overlapping regions covered more than 30% of the surface of the cell. We followed this procedure to keep the real proportion of the Brazilian Amazon territory covered by PAs. In this study, the network of the Brazilian Amazon PAs was therefore composed by 79 federal and 53 state PAs, covering approximately 24% of the biome (S1 Fig). Consequently, the proportion of grid cells assigned as “protected” (24%) is similar to the proportion of Amazon territory covered by federal and state PAs (23.5%) [25].

### Effectiveness of Protected Areas–Null model

We assessed the effectiveness of PAs by comparing the number of critically-exposed species inside each PA to the estimated number of species predicted that should be found inside the PA according to a null model. That null model randomly allocated PAs within the Amazon while retaining their size, shape and orientation (see Lemes et. al. [28] and Ferro et al. [29]). We restricted the possibilities of randomly allocated PAs to sites (cells) not covered by any PA already established. Hence, when a PA was randomly allocated to a site already covered by a PA, a new randomization run were done. In doing so, we took into account the currently established network of Amazon PAs in the randomization process. That incorporation of a realistic baseline on the randomization process has never been attempted before, to our knowledge, but adds substantial light on the predicted outcomes on PAs effectiveness in face of climate change. We used the equation below to compare the observed species richness in each PA (OR) against the species richness (RR) obtained by 1000 (n) randomization runs. A given PA was considered effective when OR ≥ RR at least 95% of runs, or p ≤ 0.05 (Eq 1)

$$p=\sum_{i=1}^{n}\frac{(RRi\geq OR)+1}{n+1}$$ (1)

## Results

**Fig 1:**
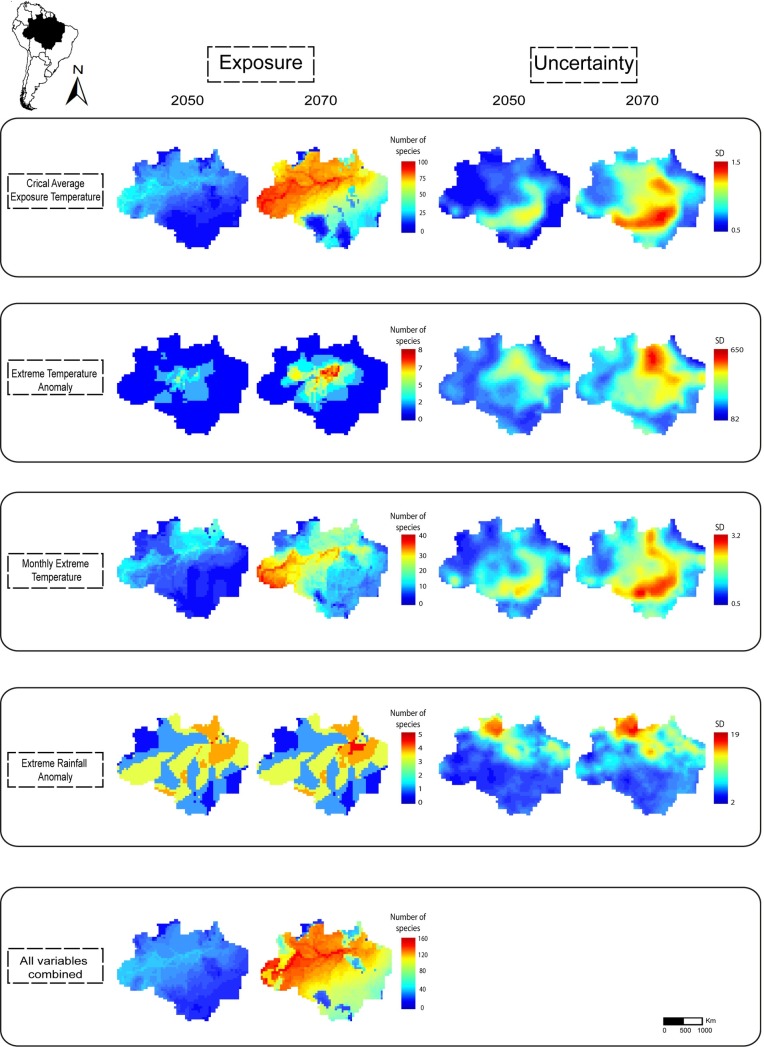
Areas in the Brazilian Amazon with highest number of critically-exposed species in relation to four climatic variables separately and taken together and uncertainty maps (standard deviation) from 15 climate models for the Brazilian Amazon. Species were considered “critically-exposed” when future climate exceeds the current climate variability for more than 80% of their range. Colors close to red indicate localities with higher number of species. These results are based on a high-emission greenhouse gases scenario (RCP8.5) for 2050 and 2070. All shapefiles are from the Brazilian Ministry of the Environment.

**Fig 2:**
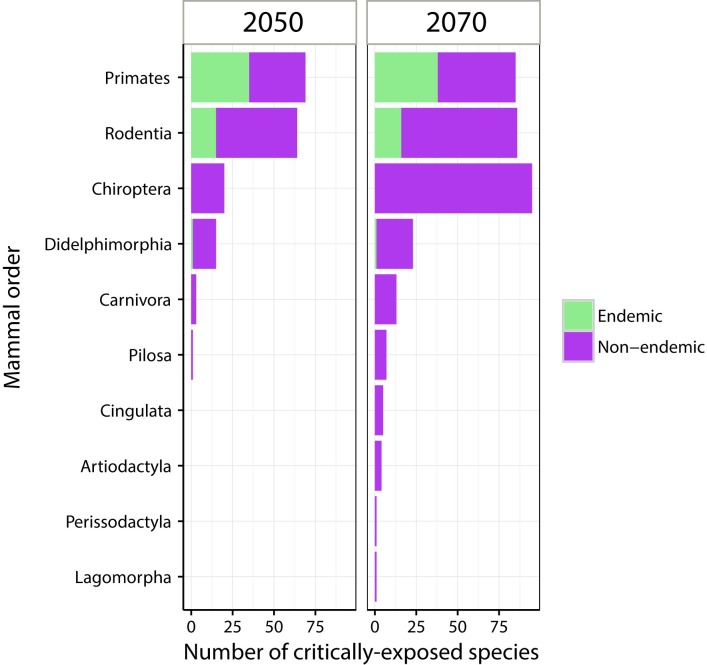
Number of critically-exposed mammals in the Brazilian Amazon exposed to four climatic variables taken together. Species were considered “critically-exposed” when future climate exceeds the current climate variability for more than 80% of their range. These results are based on a high-emission greenhouse gases scenario (RCP8.5) for 2050 and 2070. Climatic variables were mean annual temperature, temperature seasonality, maximum temperature of warmest month, and precipitation seasonality.

Our results indicated that 46% (year 2050) and 85% (year 2070) of all species were exposed to novel climate conditions in more than 80% of their ranges, although different climatic variables revealed contrasting patterns of exposure (Table 1). Overall, we found that the majority of critically-exposed species to temperature anomalies (interannual average, variability and extremes) were concentrated in western and northern Amazon (Fig 1). Critically-exposed species to rainfall anomalies were predominately concentrated in the northeastern portion of the biome (Fig 1). Few species were exposed to precipitation anomalies. However, climate variability within species’ range (maximum and minimum values) was higher for precipitation compared to temperature, which might have affected differential exposure (S2 Fig). Most of the critically-exposed species were found in low-uncertainty areas (Fig 1). Given that climatic conditions will continue to change from 2050 to 2070, our results showed that the number of critically-exposed to climate change also tend to increase (Figs 1 and 2, Table 1).

**Table 1:** Current and future climate conditions (average and standard deviation) in Brazilian Amazon. The number of critically-exposed mammals (species with more than 80% of their range exposed) exposed to different climatic variables separately and taken together are showed. These results were quantified using an average of 15 General Circulation Models for a high-emission greenhouse gases scenario (RCP 8.5) for 2050 and 2070.

	Critical Average Exposure Temperature (°C)	Extreme Temperature Anomaly (CV)	Monthly Extreme Temperature (°C)	Extreme Rainfall Anomaly (mm)	All variables combined
**Present**	25.76 ± 1.02	57.58 ± 30.07	32.84 ± 1.19	58.41 ± 17.66	-
**Future (2050)**	28.60 ± 0.97	87.63 ± 27.6	36.60 ± 1.32	63.79 ± 18.40	-
**Future (2070)**	29.95 ± 0.98	103.64 ± 27.6	38.35 ± 1.50	65.81 ± 18.8	-
**Number of species exposed (2050)**	141 (37%)	17 (4%)	96 (25%)	11 (3%)	173 (46%)
**Number of species exposed (2070)**	255 (68%)	32 (8%)	166 (44%)	12 (3%)	321 (85%)

**Fig 3:**
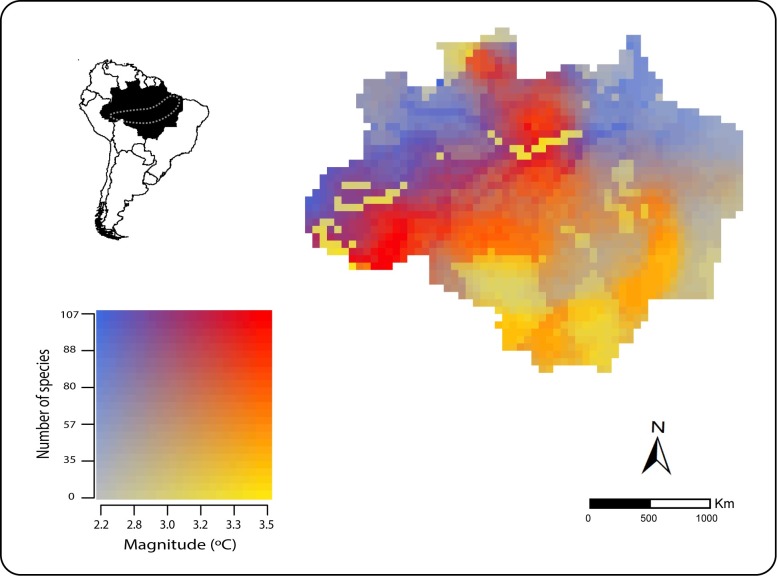
Magnitude of temperature exposure of critically-exposure mammals in the Brazilian Amazon. Areas with highest concentrations of species exposed to low and large magnitude of temperature are shown in blue and red, respectively. Areas with lowest concentration of species exposed to largest magnitude of temperature are shown in yellow. The Amazonian “arc of deforestation” is indicated in gray (dashed line). These results are based on an ensemble of 15 climate models for a high-emission greenhouse gases scenario (RCP 8.5) for 2070. All shapefiles are from the Brazilian Ministry of the Environment.

We found that 99% (54 species) of the endemic mammals analyzed in this study belonging to mammalian orders Primates (38), Rodentia (15) and Didelphimorphia (1) might be critically-exposed to climate change in 2050 (Fig 2, S2 Table). Further, some endemic and non-endemic mammals might be critically-exposed to different magnitude of increasing temperatures, ranging from 2.2°C to 3.5°C (Fig 3).

The majority of critically-exposed species were barely represented in PAs (S3 Table). Although the Amazon federal and state PAs cover around 23.5% of the Amazon [25], only 24–28% of PAs are likely to harbor more species than expected by our null model (S1 Fig). Further, the number of PAs considered effective decreased from 37 to 32 from 2050 to 2070, respectively (S3 Table).

## Discussion

In this paper, we quantified mammal local exposure to climate change and identified areas in the Brazilian Amazon with the highest concentrations of critically-exposed species. Further, we highlighted that these species are barely represented by the Amazon network of Protected Areas. Exposure was not equal along the species’ range, implying that some populations will be more at risk than others. On average, 85% of the Amazon mammals will be exposed in more than 80% of their ranges by 2070. That percentage is even higher for endemic mammals; almost all endemic species are predicted to be exposed in more than 80% of their range. The ability to identify populations at higher risk is paramount to implement proactive on-the-ground conservation actions, aiming to increase population resilience against negative effects of climate change [23,40].

However, exposure itself does not mean vulnerability. Although our results highlight a general pattern of local climate exposure in the Amazon, the overall picture of species vulnerability depends ultimately on their sensibility and adaptability to new climate conditions [4,41,42]. Studies have pointed out that some species’ ecological and physiological traits are more related to vulnerability in endotherms [41,43]. For example, larger, exclusively diurnal or nocturne mammals are more sensitive to climate change than smaller mammals and those with flexible activities periods [5,44]. Despite these findings, all else being equal, populations in places undergoing larger climatic changes are more likely to be negatively affected [23,45]. Physiology, behavior and morphology of individuals are expected to be adversely affected by reductions in climatic suitability, ultimately interfering on population dynamics [23,46]. Although exposure represents only one distal component that affect species vulnerability, it constitutes a first-order assessment to identify where species are more potentially vulnerable to climate change, particularly when data on species sensitivity and adaptive capacity are scarce [47]. Subsequently, this information can be used to complement species extinction risk assessments [23].

Species living in tropical regions are particularly vulnerable to climate change [15,16]. Tropical species tend to live closer to their maximum critical temperature (maximum temperatures that a species can withstand without additional energy expenditure to keep body temperature constant; [21]). Thus, even the slighter amount of climate change projected for the tropics might exceed species thermal tolerance. It follows that the additional amount of energy expenditure necessary to maintain homeostasis reduces energy available for other functions, such as reproduction. That decreased available energy may also lead to fitness reduction, loss of genetic variation at population level and possibly local extinction [9,48].

Among the most exposed endemic species, Primates will likely be the mammalian group most vulnerable to climate change. Besides living in areas where greater climate change is expected, Primates in the Amazon are climatic and geographically restricted and thereby expected to be more sensitive to climate change [7,17,49]. In addition, these species will probably be unable to keep pace with climate change even at relatively low magnitude of climate change due to high velocity of climate change expected in the tropics and poor dispersal abilities [50,51]. As a consequence of that high expected vulnerability, some conservation actions, such as assisted migration and *ex situ* conservation, have been proposed as potential management strategies for climate change adaptation [51].

Protected Areas are the keystone of current conservation strategies and play a vital role in buffering species against extreme exposure to climate change. PAs may be the only guarantee that species’ habitat will remaining in the future, since Amazon have been more and more fragmented [27]. Protecting species habitat, even when it may be highly exposed to climate changes, is fundamental to increase species’ resilience against climate change. PA’s support different microhabitats conditions and resources that are essential for species avoiding be high exposure, for example, behaviorally avoid overheating by moving between microhabitats [21]. Therefore, protecting species’ habitat in PA’s is fundamental to increase species resilience against climate change at the same time that avoid other synergetic threats as deforestation. In addition, our findings strengthen the need to assess the PAs effectiveness using dynamic approaches, since climate is predicted to continuously change from 2050 to 2070. Those expected changes impose new challenges for establishment of PAs or adaptation of current PAs network.

We also found that the magnitude of species exposure to climate change was uneven across the Brazilian Amazon territory. Species inhabiting south-western regions are likely to experience high magnitude of climate change in the future. Although the number of critically-exposed species may be higher at other regions, Amazon south-western species are also threatened by massive deforestation rates. That region is popularly known as the “Arc of Deforestation” [52] for its currently high rates of forest clearing. Climate change potentially interacts with deforestation and synergistic effects on biodiversity may be higher than their isolates impacts [53]. Particularly in the Amazon, deforestation and selective logging are known to create local and regional climate anomalies, for creating fire-prone warmer and drier micro-habitats. [27,54]. Large amounts of ashes and smoke released by burnings also feeds back fire susceptibility and alters water regimes [27,54]. Species inhabiting the Arch of Deforestation may therefore be exposed to the synergistic effects of climate change and deforestation and be more vulnerable than would be expected by the individual effects of those stressors.

In summary, in this work we highlight a general pattern of areas in which species with the potential to be critically-exposed to effects of climate change are concentrated. In addition, we showed that the current network of PAs has small effectiveness to protect these species. Such an examination provides the first step to a more comprehensive assessment of species vulnerability, and might guide conservation decisions on where to take place on-the-ground actions aiming at minimizing negative effects of climate change on species.

## Supporting Information

S1 Fig Spatial distribution of effective and non-effective protected areas (PAs) in the Brazilian Amazon. Effective PAs are those supposed to buffer species against effects of climate change as assessed by a null model that allocated each PA within the Amazon keeping its size, shape and orientation. These results are based on a combination of four climatic variables and a high-emission greenhouse gases scenario (RCP8.5) for 2070. See text for further details. All shapefiles are from the Brazilian Ministry of the Environment.
(TIF)

S2 Fig Current (1950–2000) climatic variability (maximum and minimum) present within all extent of 376 Amazon mammal species’ range (range not exclusively within Amazon extent). Maximum and minimum values for each climatic variable within species’ range are shown in red and green, respectively.
(TIF)

S1 Table Factor Analysis showing the collinearity among 19 bioclimatic variables. Values in bold indicate variables used to quantify mammal exposure to climate change in the Brazilian Amazon. The minus sign (-) indicates very small values not shown
(PDF)

S2 Table The 376 mammal species inhabiting the Brazilian Amazon analyzed in this study. A species was considered critically-exposed when more than 80% of its range was exposed to four climatic variables taken together. Endemic species are signed with an asterisk (*).
(PDF)

S3 Table Effectiveness of the Brazilian Amazon Protected Areas (PAs) in representing critically-exposed mammal assessed by comparing species richness of each PA against richness estimated by a null model. Federal and state PAs are subdivided into two categories: Sustainable Use (SU) and Full Protection (FP). PAs assigned with (*) were considered as effective. See methods for further details.
(PDF)
