# Supplementary figures and images for: Assessing Mammal Exposure to Climate Change in the Brazilian Amazon
Source: PLoS One. 2016 Nov 9;11(11):e0165073. doi: 10.1371/journal.pone.0165073 (PMC5102461; doi:10.1371/journal.pone.0165073)

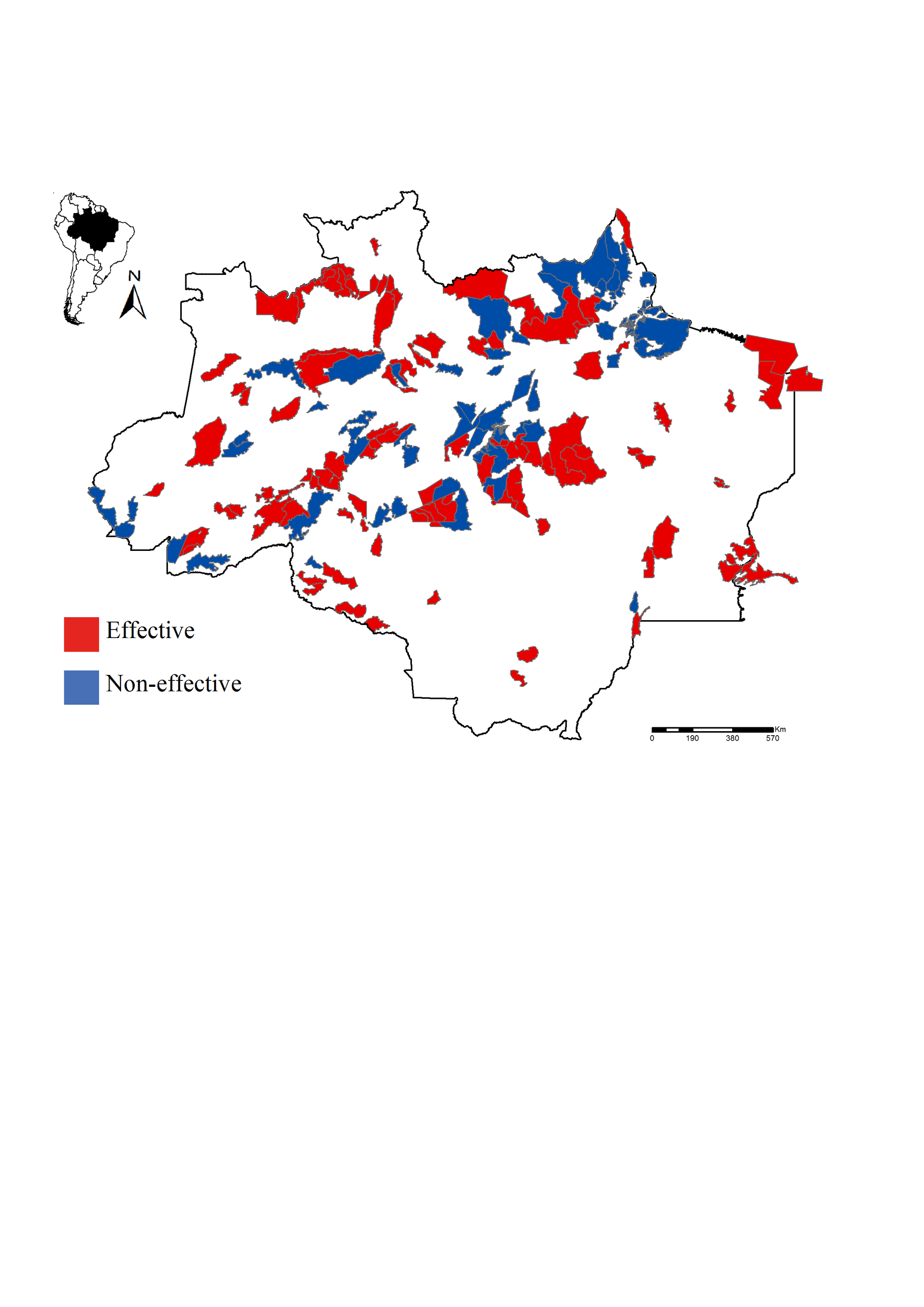

Supplement: S1 Fig — Effective PAs are those supposed to buffer species against effects of climate change as assessed by a null model that allocated each PA within the Amazon keeping its size, shape and orientation. These results are based on a combination of four climatic variables and a high-emission greenhouse gases scenario (RCP8.5) for 2070. See text for further details. All shapefiles are from the Brazilian Ministry of the Environment. (TIF) [file pone.0165073.s001.tif]

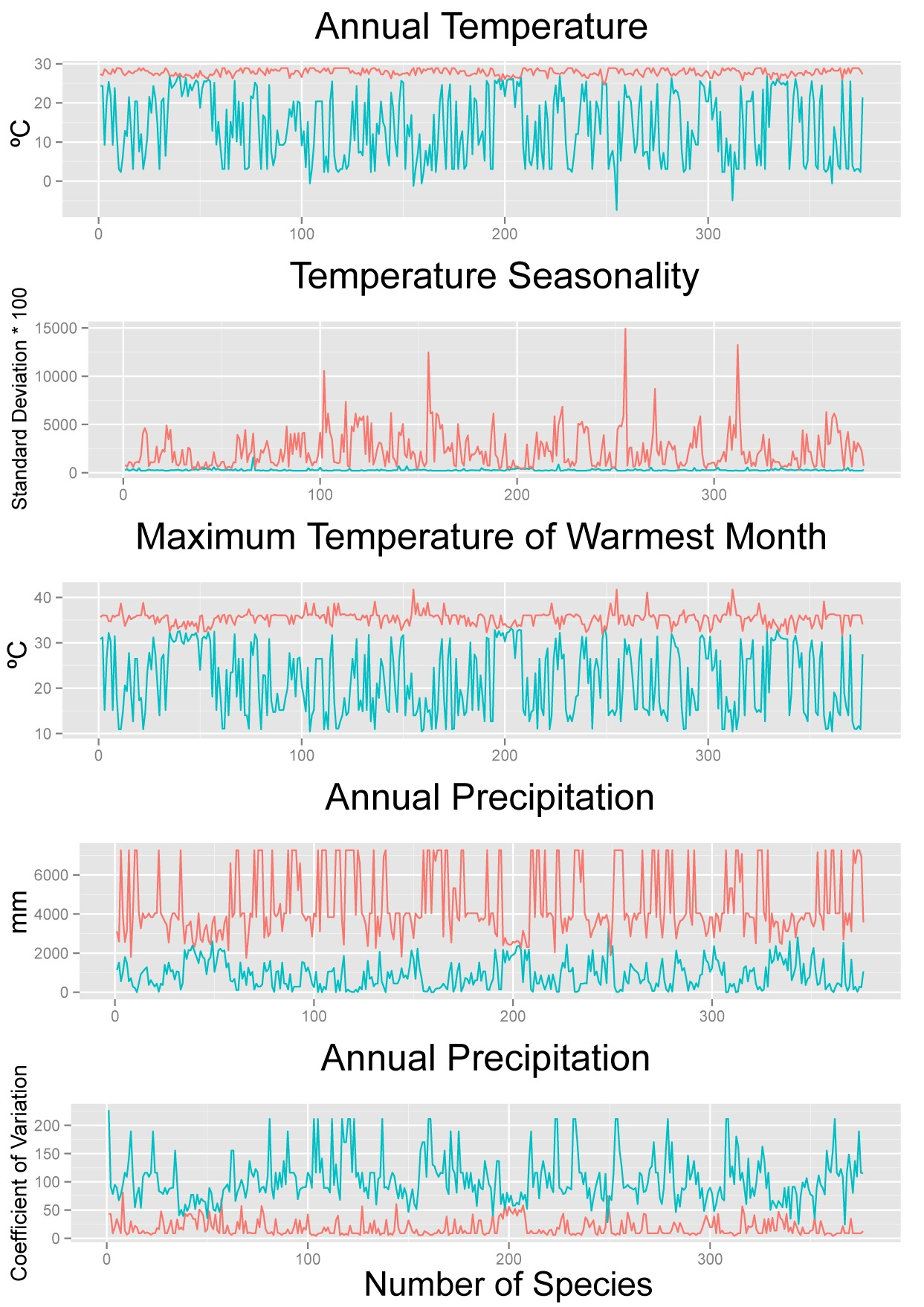

Supplement: S2 Fig — Maximum and minimum values for each climatic variable within species’ range are shown in red and green, respectively. (TIF) [file pone.0165073.s002.tif]
