# Supplementary material for: Assessing Mammal Exposure to Climate Change in the Brazilian Amazon
Source: PLoS One. 2016 Nov 9;11(11):e0165073. doi: 10.1371/journal.pone.0165073 (PMC5102461; doi:10.1371/journal.pone.0165073)
Supplement: S1 Table — Values in bold indicate variables used to quantify mammal exposure to climate change in the Brazilian Amazon. The minus sign (-) indicates very small values not shown (PDF) [file pone.0165073.s003.pdf]

S1 Table. Factor Analysis showing the collinearity among 19 bioclimatic variables. Values in bold indicate variables used to quantify mammal exposure to climate change in the Brazilian Amazon. The minus sign (-) indicates very small values not shown.

**Climatic Variables**	**Loadings**				
	Factor 1	Factor 2	Factor 3	Factor 4	Factor 5
Annual Mean Temperature	0.72	0.251	**0.641**	-	-
Mean Diurnal Range	-0.166	-0.358	-0.312	0.825	-
Isothermality	0.871	0.346	0.2	-	-
Temperature Seasonality	**-0.939**	-0.29	-0.166	-	-
Max Temperature of Warmest Month	0.229	**0.946**	0.195	-	-
Min Temperature of Coldest Month	0.836	0.277	0.454	-0.113	-
Temperature Annual Range	-0.911	-0.305	-0.149	0.217	-
Mean Temperature of Wettest Quarter	0.3	0.215	0.74	-0.13	-
Mean Temperature of Driest Quarter	0.813	0.216	0.421	-	-
Mean Temperature of Warmest Quarter	0.346	0.146	0.922	-	-
Mean Temperature of Coldest Quarter	0.832	0.271	0.48	-	-
Annual Precipitation	0.361	0.796	0.188	**0.409**	-0.137
Precipitation of Wettest Month	0.426	0.856	0.228	-0.139	-
Precipitation of Driest Month	0.32	0.934		-	-
Precipitation Seasonality	0.325	**-0.699**	0.112	-	-
Precipitation of Wettest Quarter	0.419	0.863	0.218	0.103	-0.134
Precipitation of Driest Quarter	0.115	0.36	0.919	-	-
Precipitation of Warmest Quarter	0.203	0.534	0.15	0.435	-
Precipitation of Coldest Quarter	0.253	0.632	0.392	-0.171	-
Loadings of each factor	6.054	3.792	3.582	2.845	0.92
Proportion of variance explained	0.319	0.2	0.189	0.15	0.048
Cumulative proportion of variance explained	0.319	0.518	0.707	0.856	0.905
