# Supplementary material for: Assessing Mammal Exposure to Climate Change in the Brazilian Amazon
Source: PLoS One. 2016 Nov 9;11(11):e0165073. doi: 10.1371/journal.pone.0165073 (PMC5102461; doi:10.1371/journal.pone.0165073)
Supplement: S2 Table — A species was considered critically-exposed when more than 80% of its range was exposed to four climatic variables taken together. Endemic species are signed with an asterisk (*). (PDF) [file pone.0165073.s004.pdf]

S2 Table. The 376 mammal species inhabiting the Brazilian Amazon analyzed in this study. A species was considered critically-exposed when more than 80% of its range was exposed to four climatic variables taken together. Endemic species are signed with an asterisk (*).

Species	Family	Order	Amazon species' range (n of cells)	Percentage of species range exposed		Critically-exposed species	
				**2050**	**2070**	**2050**	**2070**
Alouatta belzebul	Atelidae	Primates	318	86	100	Yes	Yes
*Alouatta discolor**	Atelidae	Primates	159	100	100	Yes	Yes
Alouatta juara	Atelidae	Primates	277	23	94	No	Yes
Alouatta macconnelli	Atelidae	Primates	344	65	86	No	Yes
*Alouatta nigerrima**	Atelidae	Primates	103	100	100	Yes	Yes
Alouatta puruensis	Atelidae	Primates	391	95	100	Yes	Yes
Alouatta seniculus	Atelidae	Primates	278	24	94	No	Yes
Alouatta ululata	Atelidae	Primates	2	100	100	Yes	Yes
Ametrida centurio	Phyllostomidae	Chiroptera	1329	44	82	No	Yes
Anoura caudifer	Phyllostomidae	Chiroptera	837	32	71	No	No
Anoura geoffroyi	Phyllostomidae	Chiroptera	279	12	46	No	No
Aotus azarae	Aotidae	Primates	788	78	96	No	Yes
Aotus nancymaae	Aotidae	Primates	103	100	100	Yes	Yes
Aotus nigriceps	Aotidae	Primates	494	97	100	Yes	Yes
Aotus trivirgatus	Aotidae	Primates	200	83	90	Yes	Yes
Aotus vociferans	Aotidae	Primates	159	86	100	Yes	Yes
Artibeus anderseni	Phyllostomidae	Chiroptera	848	98	100	Yes	Yes
Artibeus cinereus	Phyllostomidae	Chiroptera	908	66	95	No	Yes
Artibeus concolor	Phyllostomidae	Chiroptera	1318	61	95	No	Yes
Artibeus glaucus	Phyllostomidae	Chiroptera	95	20	60	No	No
Artibeus gnomus	Phyllostomidae	Chiroptera	1756	60	93	No	Yes
Artibeus lituratus	Phyllostomidae	Chiroptera	1766	17	60	No	No
Artibeus obscurus	Phyllostomidae	Chiroptera	1766	60	93	No	Yes

Species	Family	Order	Amazon species' range (n of cells)	Percentage of species range exposed		Critically-exposed species	
				**2050**	**2070**	**2050**	**2070**
Artibeus planirostris	Phyllostomidae	Chiroptera	1112	59	94	No	Yes
Ateles belzebuth	Atelidae	Primates	114	80	91	No	Yes
Ateles chamek	Atelidae	Primates	644	82	95	Yes	Yes
*Ateles marginatus**	Atelidae	Primates	203	99	100	Yes	Yes
Ateles paniscus	Atelidae	Primates	252	94	97	Yes	Yes
Atelocynus microtis	Canidae	Carnivora	716	95	100	Yes	Yes
Bassaricyon alleni	Procyonidae	Carnivora	23	91	100	Yes	Yes
Bassaricyon beddardi	Procyonidae	Carnivora	89	62	76	No	No
Bradypus tridactylus	Bradypodidae	Pilosa	206	71	99	No	Yes
Bradypus variegatus	Bradypodidae	Pilosa	1511	38	83	No	Yes
Cabassous unicinctus	Dasypodidae	Cingulata	1742	59	93	No	Yes
Cacajao ayresi	Pitheciidae	Primates	7	100	100	Yes	Yes
Cacajao calvus	Pitheciidae	Primates	65	100	100	Yes	Yes
Cacajao hosomi	Pitheciidae	Primates	19	84	100	Yes	Yes
Cacajao melanocephalus	Pitheciidae	Primates	128	92	100	Yes	Yes
*Callibella humilis**	Callitrichidae	Primates	5	100	100	Yes	Yes
*Callicebus baptista**	Pitheciidae	Primates	17	100	100	Yes	Yes
*Callicebus bernhardi**	Pitheciidae	Primates	60	100	100	Yes	Yes
Callicebus brunneus	Pitheciidae	Primates	75	100	100	Yes	Yes
*Callicebus caligatus**	Pitheciidae	Primates	39	100	100	Yes	Yes
*Callicebus cinerascens**	Pitheciidae	Primates	93	100	100	Yes	Yes
Callicebus cupreus	Pitheciidae	Primates	248	100	100	Yes	Yes
Callicebus donacophilus	Pitheciidae	Primates	16	100	100	Yes	Yes
*Callicebus dubius**	Pitheciidae	Primates	61	100	100	Yes	Yes
*Callicebus hoffmannsi**	Pitheciidae	Primates	47	100	100	Yes	Yes
Callicebus lucifer	Pitheciidae	Primates	41	100	100	Yes	Yes
Callicebus lugens	Pitheciidae	Primates	139	70	88	No	Yes
*Callicebus moloch**	Pitheciidae	Primates	348	93	100	Yes	Yes
*Callicebus purinus**	Pitheciidae	Primates	72	100	100	Yes	Yes

Species	Family	Order	Amazon species' range (n of cells)	Percentage of species range exposed		Critically-exposed species	
				**2050**	**2070**	**2050**	**2070**
*Callicebus regulus**	Pitheciidae	Primates	97	100	100	Yes	Yes
*Callicebus stephennashi**	Pitheciidae	Primates	21	100	100	Yes	Yes
*Callicebus torquatus**	Pitheciidae	Primates	110	100	100	Yes	Yes
Callimico goeldii	Callitrichidae	Primates	88	100	100	Yes	Yes
Calomys tocantinsi	Cricetidae	Rodentia	55	100	100	Yes	Yes
Caluromys lanatus	Didelphidae	Didelphimorphia	507	26	90	No	Yes
Caluromys philander	Didelphidae	Didelphimorphia	954	71	94	No	Yes
Caluromysiops irrupta	Didelphidae	Didelphimorphia	32	100	100	Yes	Yes
Carollia brevicauda	Phyllostomidae	Chiroptera	1662	42	82	No	Yes
Carollia perspicillata	Phyllostomidae	Chiroptera	1766	37	80	No	Yes
Cebuella pygmaea	Callitrichidae	Primates	353	98	100	Yes	Yes
Cebus albifrons	Cebidae	Primates	684	61	99	No	Yes
Cebus apella	Cebidae	Primates	910	78	96	No	Yes
Cebus cay	Cebidae	Primates	17	100	100	Yes	Yes
*Cebus kaapori**	Cebidae	Primates	83	100	100	Yes	Yes
Cebus macrocephalus	Cebidae	Primates	471	53	100	No	Yes
Cebus olivaceus	Cebidae	Primates	270	60	85	No	Yes
Centronycteris centralis	Emballonuridae	Chiroptera	46	50	85	No	Yes
Centronycteris maximiliani	Emballonuridae	Chiroptera	1262	86	98	Yes	Yes
Chiroderma trinitatum	Phyllostomidae	Chiroptera	1727	39	81	No	Yes
Chiroderma villosum	Phyllostomidae	Chiroptera	1699	36	80	No	No
Chironectes minimus	Didelphidae	Didelphimorphia	403	18	67	No	No
*Chiropotes albinasus**	Pitheciidae	Primates	372	80	93	No	Yes
*Chiropotes chiropotes**	Pitheciidae	Primates	323	65	85	No	Yes
*Chiropotes satanas**	Pitheciidae	Primates	114	100	100	Yes	Yes
*Chiropotes utahickae**	Pitheciidae	Primates	149	78	100	No	Yes
Choeroniscus godmani	Phyllostomidae	Chiroptera	86	30	56	No	No
Choeroniscus minor	Phyllostomidae	Chiroptera	1132	60	94	No	Yes
Choloepus didactylus	Megalonychidae	Pilosa	889	80	97	No	Yes

Species	Family	Order	Amazon species' range (n of cells)	Percentage of species range exposed		Critically-exposed species	
				**2050**	**2070**	**2050**	**2070**
Choloepus hoffmanni	Megalonychidae	Pilosa	128	98	100	Yes	Yes
Chrotopterus auritus	Phyllostomidae	Chiroptera	1766	52	85	No	Yes
*Coendou nycthemera**	Erethizontidae	Rodentia	174	100	100	Yes	Yes
Coendou prehensilis	Erethizontidae	Rodentia	1692	53	89	No	Yes
Cormura brevirostris	Emballonuridae	Chiroptera	1604	59	93	No	Yes
Cuniculus paca	Cuniculidae	Rodentia	1752	47	87	No	Yes
Cyclopes didactylus	Cyclopedidae	Pilosa	1406	59	94	No	Yes
Cynomops abrasus	Molossidae	Chiroptera	1766	61	93	No	Yes
Cynomops greenhalli	Molossidae	Chiroptera	600	38	77	No	No
Cynomops paranus	Molossidae	Chiroptera	1746	40	82	No	Yes
Cynomops planirostris	Molossidae	Chiroptera	1766	40	82	No	Yes
Cyttarops alecto	Emballonuridae	Chiroptera	155	10	65	No	No
Dactylomys boliviensis	Echimyidae	Rodentia	42	100	100	Yes	Yes
Dactylomys dactylinus	Echimyidae	Rodentia	992	91	100	Yes	Yes
Dasyprocta cristata	Dasyproctidae	Rodentia	85	86	93	Yes	Yes
Dasyprocta fuliginosa	Dasyproctidae	Rodentia	458	40	95	No	Yes
Dasyprocta leporina	Dasyproctidae	Rodentia	375	66	86	No	Yes
Dasyprocta prymnolopha	Dasyproctidae	Rodentia	201	90	100	Yes	Yes
Dasyprocta punctata	Dasyproctidae	Rodentia	49	96	100	Yes	Yes
Dasypus kappleri	Dasypodidae	Cingulata	1204	56	92	No	Yes
Dasypus novemcinctus	Dasypodidae	Cingulata	1766	17	60	No	No
Dasypus septemcinctus	Dasypodidae	Cingulata	736	72	94	No	Yes
Desmodus rotundus	Phyllostomidae	Chiroptera	1766	34	79	No	No
Diaemus youngi	Phyllostomidae	Chiroptera	1766	38	81	No	Yes
Diclidurus albus	Emballonuridae	Chiroptera	1766	17	60	No	No
Diclidurus ingens	Emballonuridae	Chiroptera	353	57	92	No	Yes
Diclidurus isabellus	Emballonuridae	Chiroptera	255	84	89	Yes	Yes
Diclidurus scutatus	Emballonuridae	Chiroptera	714	82	97	Yes	Yes
Didelphis imperfecta	Didelphidae	Didelphimorphia	48	19	40	No	No

Species	Family	Order	Amazon species' range (n of cells)	Percentage of species range exposed		Critically-exposed species	
				**2050**	**2070**	**2050**	**2070**
Didelphis marsupialis	Didelphidae	Didelphimorphia	1423	36	80	No	No
Dinomys branickii	Dinomyidae	Rodentia	280	54	100	No	Yes
Diphylla ecaudata	Phyllostomidae	Chiroptera	631	6	48	No	No
Echimys chrysurus	Echimyidae	Rodentia	421	95	98	Yes	Yes
*Echimys vieirai**	Echimyidae	Rodentia	34	100	100	Yes	Yes
Eira barbara	Mustelidae	Carnivora	1766	49	82	No	Yes
Eptesicus brasiliensis	Vespertilionidae	Chiroptera	1766	40	82	No	Yes
Eptesicus furinalis	Vespertilionidae	Chiroptera	1766	17	60	No	No
Eumops auripendulus	Molossidae	Chiroptera	1766	38	81	No	Yes
Eumops bonariensis	Molossidae	Chiroptera	1766	34	79	No	No
Eumops dabbenei	Molossidae	Chiroptera	18	17	83	No	Yes
Eumops glaucinus	Molossidae	Chiroptera	1431	43	82	No	Yes
Eumops hansae	Molossidae	Chiroptera	1046	36	80	No	No
Eumops perotis	Molossidae	Chiroptera	1557	78	96	No	Yes
Eumops trumbulli	Molossidae	Chiroptera	1142	59	94	No	Yes
Euphractus sexcinctus	Dasypodidae	Cingulata	870	69	93	No	Yes
*Euryoryzomys emmonsae**	Cricetidae	Rodentia	108	79	100	No	Yes
Euryoryzomys macconnelli	Cricetidae	Rodentia	968	82	98	Yes	Yes
Euryoryzomys nitidus	Cricetidae	Rodentia	130	98	100	Yes	Yes
Furipterus horrens	Furipteridae	Chiroptera	1438	50	84	No	Yes
Galea spixii	Caviidae	Rodentia	340	72	93	No	Yes
Galictis vittata	Mustelidae	Carnivora	1691	50	88	No	Yes
Glironia venusta	Didelphidae	Didelphimorphia	42	100	100	Yes	Yes
Glossophaga commissarisi	Phyllostomidae	Chiroptera	372	31	89	No	Yes
Glossophaga longirostris	Phyllostomidae	Chiroptera	72	26	54	No	No
Glossophaga soricina	Phyllostomidae	Chiroptera	1766	17	59	No	No
Glyphonycteris daviesi	Phyllostomidae	Chiroptera	1028	60	96	No	Yes
Glyphonycteris sylvestris	Phyllostomidae	Chiroptera	1056	46	88	No	Yes
Gracilinanus emiliae	Didelphidae	Didelphimorphia	60	98	98	Yes	Yes

Species	Family	Order	Amazon species' range (n of cells)	Percentage of species range exposed		Critically-exposed species	
				2050	2070	2050	2070
Histiotus velatus	Vespertilionidae	Chiroptera	35	77	94	No	Yes
Holochilus sciureus	Cricetidae	Rodentia	1766	60	93	No	Yes
Hydrochoerus hydrochaeris	Caviidae	Rodentia	1766	60	93	No	Yes
Hyladelphys kalinowskii	Didelphidae	Didelphimorphia	648	94	100	Yes	Yes
Hylaeamys acritus	Cricetidae	Rodentia	15	87	100	Yes	Yes
Hylaeamys megacephalus	Cricetidae	Rodentia	1283	81	95	Yes	Yes
Hylaeamys perenensis	Cricetidae	Rodentia	412	97	100	Yes	Yes
Hylaeamys yunganus	Cricetidae	Rodentia	1172	87	98	Yes	Yes
Isothrix bistriata	Echimyidae	Rodentia	332	88	96	Yes	Yes
*Isothrix negrensis**	Echimyidae	Rodentia	121	100	100	Yes	Yes
*Isothrix pagurus**	Echimyidae	Rodentia	138	100	100	Yes	Yes
Lagothrix cana	Atelidae	Primates	436	93	100	Yes	Yes
Lagothrix lagotricha	Atelidae	Primates	78	71	100	No	Yes
Lagothrix poeppigii	Atelidae	Primates	109	100	100	Yes	Yes
Lampronycteris brachyotis	Phyllostomidae	Chiroptera	763	89	97	Yes	Yes
Lasiurus blossevillii	Vespertilionidae	Chiroptera	1766	17	59	No	No
Lasiurus cinereus	Vespertilionidae	Chiroptera	224	60	87	No	Yes
Lasiurus ega	Vespertilionidae	Chiroptera	1766	17	60	No	No
Lasiurus egregius	Vespertilionidae	Chiroptera	28	4	43	No	No
Leopardus colocolo	Felidae	Carnivora	175	67	93	No	Yes
Leopardus pardalis	Felidae	Carnivora	1749	31	76	No	No
Leopardus tigrinus	Felidae	Carnivora	1766	60	93	No	Yes
Leopardus wiedii	Felidae	Carnivora	1766	24	66	No	No
Lichonycteris obscura	Phyllostomidae	Chiroptera	1282	48	87	No	Yes
Lionycteris spurrelli	Phyllostomidae	Chiroptera	1310	32	80	No	Yes
Lonchophylla mordax	Phyllostomidae	Chiroptera	150	92	99	Yes	Yes
Lonchophylla thomasi	Phyllostomidae	Chiroptera	1476	35	79	No	No
Lonchorhina aurita	Phyllostomidae	Chiroptera	1765	40	82	No	Yes
Lonchorhina inusitata	Phyllostomidae	Chiroptera	758	86	97	Yes	Yes

Species	Family	Order	Amazon species' range (n of cells)	Percentage of species range exposed		Critically-exposed species	
				**2050**	**2070**	**2050**	**2070**
*Lonchothrix emiliae**	Echimyidae	Rodentia	152	99	100	Yes	Yes
Lontra longicaudis	Mustelidae	Carnivora	1752	27	66	No	No
Lophostoma brasiliense	Phyllostomidae	Chiroptera	1250	31	80	No	No
Lophostoma carrikeri	Phyllostomidae	Chiroptera	1096	58	94	No	Yes
Lophostoma schulzi	Phyllostomidae	Chiroptera	107	94	100	Yes	Yes
Lophostoma silvicolum	Phyllostomidae	Chiroptera	1766	43	82	No	Yes
Macrophyllum macrophyllum	Phyllostomidae	Chiroptera	1766	38	81	No	Yes
Makalata didelphoides	Echimyidae	Rodentia	890	63	89	No	Yes
Makalata macrura	Echimyidae	Rodentia	554	99	100	Yes	Yes
Marmosa demerarae	Didelphidae	Didelphimorphia	1626	64	93	No	Yes
Marmosa lepida	Didelphidae	Didelphimorphia	754	59	95	No	Yes
Marmosa murina	Didelphidae	Didelphimorphia	1240	62	95	No	Yes
Marmosa regina	Didelphidae	Didelphimorphia	157	100	100	Yes	Yes
Marmosops bishopi	Didelphidae	Didelphimorphia	265	66	89	No	Yes
Marmosops neblina	Didelphidae	Didelphimorphia	165	99	100	Yes	Yes
Marmosops noctivagus	Didelphidae	Didelphimorphia	442	98	100	Yes	Yes
Marmosops parvidens	Didelphidae	Didelphimorphia	282	95	98	Yes	Yes
Marmosops pinheiroi	Didelphidae	Didelphimorphia	111	95	95	Yes	Yes
Mazama americana	Cervidae	Artiodactyla	1707	40	81	No	Yes
Mazama gouazoubira	Cervidae	Artiodactyla	205	65	89	No	Yes
Mazama nemorivaga	Cervidae	Artiodactyla	1608	50	88	No	Yes
Mesomys hispidus	Echimyidae	Rodentia	995	82	97	Yes	Yes
*Mesomys occultus**	Echimyidae	Rodentia	27	100	100	Yes	Yes
*Mesomys stimulax**	Echimyidae	Rodentia	174	100	100	Yes	Yes
Mesophylla macconnelli	Phyllostomidae	Chiroptera	1610	38	80	No	Yes
Metachirus nudicaudatus	Didelphidae	Didelphimorphia	1638	49	87	No	Yes
*Mico acariensis**	Callitrichidae	Primates	15	100	100	Yes	Yes
*Mico argentatus**	Callitrichidae	Primates	64	100	100	Yes	Yes
*Mico chrysoleucus**	Callitrichidae	Primates	24	100	100	Yes	Yes

Species	Family	Order	Amazon species' range (n of cells)	Percentage of species range exposed		Critically-exposed species	
				2050	2070	2050	2070
*Mico emiliae**	Callitrichidae	Primates	76	100	100	Yes	Yes
*Mico humeralifer**	Callitrichidae	Primates	33	100	100	Yes	Yes
*Mico intermedius**	Callitrichidae	Primates	35	100	100	Yes	Yes
*Mico leucippe**	Callitrichidae	Primates	10	100	100	Yes	Yes
*Mico manicorensis**	Cebidae	Primates	15	100	100	Yes	Yes
*Mico marcai**	Callitrichidae	Primates	7	100	100	Yes	Yes
*Mico mauesi**	Callitrichidae	Primates	21	100	100	Yes	Yes
Mico melanurus	Callitrichidae	Primates	180	100	100	Yes	Yes
*Mico nigriceps**	Callitrichidae	Primates	18	100	100	Yes	Yes
*Mico rondoni**	Callitrichidae	Primates	37	100	100	Yes	Yes
*Mico saterei**	Callitrichidae	Primates	16	100	100	Yes	Yes
Micronycteris hirsuta	Phyllostomidae	Chiroptera	1389	69	90	No	Yes
Micronycteris megalotis	Phyllostomidae	Chiroptera	1766	40	82	No	Yes
Micronycteris microtis	Phyllostomidae	Chiroptera	1047	62	95	No	Yes
Micronycteris minuta	Phyllostomidae	Chiroptera	1765	50	88	No	Yes
Micronycteris schmidtorum	Phyllostomidae	Chiroptera	1041	45	88	No	Yes
Microsciurus flaviventer	Sciuridae	Rodentia	279	100	100	Yes	Yes
Mimon bennettii	Phyllostomidae	Chiroptera	295	67	84	No	Yes
Mimon crenulatum	Phyllostomidae	Chiroptera	1704	39	81	No	Yes
Molossops mattogrossensis	Molossidae	Chiroptera	1556	64	95	No	Yes
Molossops neglectus	Molossidae	Chiroptera	1081	59	94	No	Yes
Molossops temminckii	Molossidae	Chiroptera	1551	57	93	No	Yes
Molossus coibensis	Molossidae	Chiroptera	869	57	90	No	Yes
Molossus currentium	Molossidae	Chiroptera	100	78	93	No	Yes
Molossus molossus	Molossidae	Chiroptera	1766	38	80	No	No
Molossus rufus	Molossidae	Chiroptera	1766	17	60	No	No
Monodelphis brevicaudata	Didelphidae	Didelphimorphia	407	77	94	No	Yes
Monodelphis emiliae	Didelphidae	Didelphimorphia	420	98	100	Yes	Yes
Monodelphis glirina	Didelphidae	Didelphimorphia	367	88	100	Yes	Yes

Species	Family	Order	Amazon species' range (n of cells)	Percentage of species range exposed		Critically-exposed species	
				**2050**	**2070**	**2050**	**2070**
*Monodelphis maraxina**	Didelphidae	Didelphimorphia	20	100	100	Yes	Yes
Mustela africana	Mustelidae	Carnivora	1310	81	97	Yes	Yes
Myoprocta acouchy	Dasyproctidae	Rodentia	248	96	99	Yes	Yes
Myoprocta pratti	Dasyproctidae	Rodentia	514	65	98	No	Yes
Myotis albescens	Vespertilionidae	Chiroptera	1766	36	80	No	Yes
Myotis nigricans	Vespertilionidae	Chiroptera	1766	34	79	No	No
Myotis riparius	Vespertilionidae	Chiroptera	1766	40	82	No	Yes
Myotis simus	Vespertilionidae	Chiroptera	829	89	100	Yes	Yes
Myrmecophaga tridactyla	Myrmecophagidae	Pilosa	1758	45	86	No	Yes
Nasua nasua	Procyonidae	Carnivora	1766	63	94	No	Yes
Natalus espiritosantensis	Natalidae	Chiroptera	345	77	100	No	Yes
Neacomys dubosti	Cricetidae	Rodentia	60	100	100	Yes	Yes
Neacomys guianae	Cricetidae	Rodentia	28	75	75	No	No
*Neacomys minutus**	Cricetidae	Rodentia	84	100	100	Yes	Yes
Neacomys musseri	Cricetidae	Rodentia	32	100	100	Yes	Yes
Neacomys paracou	Cricetidae	Rodentia	208	93	97	Yes	Yes
Neacomys spinosus	Cricetidae	Rodentia	481	80	96	Yes	Yes
Necromys lasiurus	Cricetidae	Rodentia	585	65	90	No	Yes
Necromys urichi	Cricetidae	Rodentia	35	29	66	No	No
Nectomys apicalis	Cricetidae	Rodentia	174	99	100	Yes	Yes
Nectomys rattus	Cricetidae	Rodentia	1536	71	94	No	Yes
Neonycteris pusilla	Phyllostomidae	Chiroptera	6	100	100	Yes	Yes
*Neusticomys ferreirai**	Cricetidae	Rodentia	2	100	100	Yes	Yes
Neusticomys oyapocki	Cricetidae	Rodentia	23	100	100	Yes	Yes
Noctilio albiventris	Noctilionidae	Chiroptera	1766	57	87	No	Yes
Noctilio leporinus	Noctilionidae	Chiroptera	1766	17	60	No	No
Nyctinomops laticaudatus	Molossidae	Chiroptera	1696	41	82	No	Yes
Nyctinomops macrotis	Molossidae	Chiroptera	1766	40	82	No	Yes
Odocoileus virginianus	Cervidae	Artiodactyla	124	52	69	No	No

Species	Family	Order	Amazon species' range (n of cells)	Percentage of species range exposed		Critically-exposed species	
				**2050**	**2070**	**2050**	**2070**
Oecomys auyantepui	Cricetidae	Rodentia	270	94	97	Yes	Yes
Oecomys bicolor	Cricetidae	Rodentia	1621	50	87	No	Yes
Oecomys concolor	Cricetidae	Rodentia	1168	49	91	No	Yes
*Oecomys paricola**	Cricetidae	Rodentia	160	100	100	Yes	Yes
Oecomys rex	Cricetidae	Rodentia	71	93	99	Yes	Yes
Oecomys roberti	Cricetidae	Rodentia	1209	78	95	No	Yes
Oecomys rutilus	Cricetidae	Rodentia	311	91	96	Yes	Yes
Oecomys superans	Cricetidae	Rodentia	217	100	100	Yes	Yes
Oecomys trinitatis	Cricetidae	Rodentia	405	78	94	No	Yes
Oligoryzomys fulvescens	Cricetidae	Rodentia	153	41	65	No	No
Oligoryzomys microtis	Cricetidae	Rodentia	691	81	96	Yes	Yes
*Oxymycterus amazonicus**	Cricetidae	Rodentia	305	99	100	Yes	Yes
Oxymycterus inca	Cricetidae	Rodentia	4	100	100	Yes	Yes
Panthera onca	Felidae	Carnivora	1305	58	93	No	Yes
Pecari tajacu	Tayassuidae	Artiodactyla	1766	17	59	No	No
Peropteryx kappleri	Emballonuridae	Chiroptera	1759	38	81	No	Yes
Peropteryx leucoptera	Emballonuridae	Chiroptera	1154	84	98	Yes	Yes
Peropteryx macrotis	Emballonuridae	Chiroptera	1766	37	80	No	Yes
Philander andersoni	Didelphidae	Didelphimorphia	183	82	94	Yes	Yes
Philander mcilhennyi	Didelphidae	Didelphimorphia	164	100	100	Yes	Yes
Philander opossum	Didelphidae	Didelphimorphia	1383	88	98	Yes	Yes
Phylloderma stenops	Phyllostomidae	Chiroptera	1580	38	81	No	Yes
Phyllostomus discolor	Phyllostomidae	Chiroptera	1766	38	81	No	Yes
Phyllostomus elongatus	Phyllostomidae	Chiroptera	1747	60	93	No	Yes
Phyllostomus hastatus	Phyllostomidae	Chiroptera	1766	40	82	No	Yes
Phyllostomus latifolius	Phyllostomidae	Chiroptera	353	82	94	Yes	Yes
*Pithecia albicans**	Pitheciidae	Primates	34	100	100	Yes	Yes
Pithecia irrorata	Pitheciidae	Primates	458	100	100	Yes	Yes
Pithecia monachus	Pitheciidae	Primates	139	100	100	Yes	Yes

Species	Family	Order	Amazon species' range (n of cells)	Percentage of species range exposed		Critically-exposed species	
				2050	2070	2050	2070
Pithecia pithecia	Pitheciidae	Primates	258	97	100	Yes	Yes
Platyrrhinus aurarius	Phyllostomidae	Chiroptera	39	21	36	No	No
Platyrrhinus brachycephalus	Phyllostomidae	Chiroptera	1130	57	92	No	Yes
Platyrrhinus helleri	Phyllostomidae	Chiroptera	1734	21	71	No	No
Platyrrhinus infuscus	Phyllostomidae	Chiroptera	39	82	100	Yes	Yes
Platyrrhinus lineatus	Procyonidae	Carnivora	303	60	91	No	Yes
Potos flavus	Dasypodidae	Cingulata	1766	40	82	No	Yes
Priodontes maximus	Procyonidae	Carnivora	1721	59	93	No	Yes
Procyon cancrivorus	Echimyidae	Rodentia	1755	34	80	No	No
Proechimys brevicauda	Echimyidae	Rodentia	166	96	100	Yes	Yes
Proechimys cuvieri	Echimyidae	Rodentia	825	89	97	Yes	Yes
Proechimys echinothrix	Echimyidae	Rodentia	114	100	100	Yes	Yes
Proechimys gardneri	Echimyidae	Rodentia	136	100	100	Yes	Yes
*Proechimys goeldii**	Echimyidae	Rodentia	382	92	100	Yes	Yes
Proechimys guyannensis	Echimyidae	Rodentia	301	63	86	No	Yes
Proechimys hoplomyoides	Echimyidae	Rodentia	38	21	50	No	No
Proechimys kulinae	Echimyidae	Rodentia	39	100	100	Yes	Yes
Proechimys pattoni	Echimyidae	Rodentia	32	100	100	Yes	Yes
Proechimys quadruplicatus	Echimyidae	Rodentia	242	65	91	No	Yes
Proechimys roberti	Echimyidae	Rodentia	552	87	100	Yes	Yes
Proechimys simonsi	Echimyidae	Rodentia	312	99	100	Yes	Yes
Proechimys steerei	Molossidae	Chiroptera	465	96	100	Yes	Yes
Promops centralis	Molossidae	Chiroptera	44	61	89	No	Yes
Promops nasutus	Mormoopidae	Chiroptera	741	52	93	No	Yes
Pteronotus gymnonotus	Mormoopidae	Chiroptera	272	17	71	No	No
Pteronotus personatus	Mustelidae	Carnivora	389	9	37	No	No
Pteronura brasiliensis	Felidae	Carnivora	1563	59	92	No	Yes
Puma concolor	Felidae	Carnivora	1766	17	59	No	No
Puma yagouaroundi	Phyllostomidae	Chiroptera	1766	24	66	No	No

Species	Family	Order	Amazon species' range (n of cells)	Percentage of species range exposed		Critically-exposed species	
				2050	2070	2050	2070
Rhinophylla fischerae	Phyllostomidae	Chiroptera	997	63	97	No	Yes
Rhinophylla pumilio	Cricetidae	Rodentia	1446	60	95	No	Yes
*Rhipidomys emiliae**	Cricetidae	Rodentia	274	86	100	Yes	Yes
Rhipidomys gardneri	Cricetidae	Rodentia	28	100	100	Yes	Yes
Rhipidomys leucodactylus	Cricetidae	Rodentia	768	83	97	Yes	Yes
Rhipidomys macconnelli	Cricetidae	Rodentia	30	13	47	No	No
Rhipidomys nitela	Cricetidae	Rodentia	265	67	91	No	Yes
Rhipidomys wetzeli	Vespertilionidae	Chiroptera	28	18	54	No	No
Rhogeessa hussoni	Vespertilionidae	Chiroptera	158	98	100	Yes	Yes
Rhogeessa io	Emballonuridae	Chiroptera	1235	60	88	No	Yes
Rhynchonycteris naso	Emballonuridae	Chiroptera	1764	38	81	No	Yes
Saccopteryx bilineata	Emballonuridae	Chiroptera	1766	17	60	No	No
Saccopteryx canescens	Emballonuridae	Chiroptera	1171	32	78	No	No
Saccopteryx gymnura	Emballonuridae	Chiroptera	189	95	99	Yes	Yes
Saccopteryx leptura	Callitrichidae	Primates	1765	38	81	No	Yes
*Saguinus bicolor**	Callitrichidae	Primates	11	100	100	Yes	Yes
Saguinus fuscicollis	Callitrichidae	Primates	361	100	100	Yes	Yes
Saguinus imperator	Callitrichidae	Primates	70	100	100	Yes	Yes
Saguinus inustus	Callitrichidae	Primates	90	87	100	Yes	Yes
Saguinus labiatus	Callitrichidae	Primates	133	100	100	Yes	Yes
*Saguinus martinsi**	Callitrichidae	Primates	23	100	100	Yes	Yes
Saguinus melanoleucus	Callitrichidae	Primates	30	100	100	Yes	Yes
Saguinus midas	Callitrichidae	Primates	221	98	100	Yes	Yes
Saguinus mystax	Callitrichidae	Primates	189	100	100	Yes	Yes
*Saguinus niger**	Callitrichidae	Primates	232	100	100	Yes	Yes
Saguinus nigricollis	Cebidae	Primates	13	100	100	Yes	Yes
Saimiri boliviensis	Cebidae	Primates	224	100	100	Yes	Yes
Saimiri sciureus	Cebidae	Primates	1078	63	95	No	Yes
*Saimiri ustus**	Cebidae	Primates	341	90	98	Yes	Yes

Species	Family	Order	Amazon species' range (n of cells)	Percentage of species range exposed		Critically-exposed species	
				**2050**	**2070**	**2050**	**2070**
*Saimiri vanzolinii**	Sciuridae	Rodentia	4	100	100	Yes	Yes
Sciurillus pusillus	Sciuridae	Rodentia	208	100	100	Yes	Yes
Sciurus aestuans	Sciuridae	Rodentia	1220	85	97	Yes	Yes
Sciurus gilvigularis	Sciuridae	Rodentia	430	82	98	Yes	Yes
Sciurus ignitus	Sciuridae	Rodentia	107	98	100	Yes	Yes
Sciurus igniventris	Sciuridae	Rodentia	455	44	94	No	Yes
Sciurus spadiceus	Phyllostomidae	Chiroptera	616	83	98	Yes	Yes
Scleronycteris ega	Cricetidae	Rodentia	195	84	95	Yes	Yes
Scolomys ucayalensis	Cricetidae	Rodentia	44	100	100	Yes	Yes
Sigmodon alstoni	Family	Rodentia	141	100	100	Yes	Yes
Speothos venaticus	Canidae	Carnivora	1766	50	88	No	Yes
Sphaeronycteris toxophyllum	Phyllostomidae	Chiroptera	762	41	90	No	Yes
Sphiggurus melanurus	Erethizontidae	Rodentia	313	88	94	Yes	Yes
Sphiggurus roosmalenorum	Erethizontidae	Rodentia	75	96	100	Yes	Yes
Sturnira lilium	Phyllostomidae	Chiroptera	1766	29	66	No	No
Sturnira magna	Phyllostomidae	Chiroptera	30	73	100	No	Yes
Sturnira tildae	Phyllostomidae	Chiroptera	1752	59	93	No	Yes
Sylvilagus brasiliensis	Leporidae	Lagomorpha	1008	50	92	No	Yes
Tadarida brasiliensis	Molossidae	Chiroptera	10	10	40	No	No
Tamandua tetradactyla	Myrmecophagidae	Pilosa	1766	56	92	No	Yes
Tapirus terrestris	Tapiridae	Perissodactyla	1766	60	93	No	Yes
Tayassu pecari	Tayassuidae	Artiodactyla	1766	60	93	No	Yes
Thyroptera discifera	Thyropteridae	Chiroptera	983	34	78	No	No
Thyroptera lavali	Thyropteridae	Chiroptera	1	100	100	Yes	Yes
Thyroptera tricolor	Thyropteridae	Chiroptera	1458	94	99	Yes	Yes
Tonatia bidens	Phyllostomidae	Chiroptera	76	51	88	No	Yes
Tonatia saurophila	Phyllostomidae	Chiroptera	690	33	75	No	No
*Toromys grandis**	Echimyidae	Rodentia	96	100	100	Yes	Yes
Trachops cirrhosus	Phyllostomidae	Chiroptera	1761	41	81	No	Yes

Species	Family	Order	Amazon species' range (n of cells)	Percentage of species range exposed		Critically-exposed species	
				**2050**	**2070**	**2050**	**2070**
Trinycteris nicefori	Phyllostomidae	Chiroptera	390	14	63	No	No
Uroderma bilobatum	Phyllostomidae	Chiroptera	1761	38	81	No	Yes
Uroderma magnirostrum	Phyllostomidae	Chiroptera	1762	40	82	No	Yes
Vampyressa bidens	Phyllostomidae	Chiroptera	1254	57	94	No	Yes
Vampyressa brocki	Phyllostomidae	Chiroptera	235	97	99	Yes	Yes
