# Supplementary material for: Assessing Mammal Exposure to Climate Change in the Brazilian Amazon
Source: PLoS One. 2016 Nov 9;11(11):e0165073. doi: 10.1371/journal.pone.0165073 (PMC5102461; doi:10.1371/journal.pone.0165073)
Supplement: S3 Table — Federal and state PAs are subdivided into two categories: Sustainable Use (SU) and Full Protection (FP). PAs assigned with (*) were considered as effective. See methods for further details. (PDF) [file pone.0165073.s005.pdf]

S3 Table. Effectiveness of the Brazilian Amazon Protected Areas (PAs) in representing critically-exposed mammal assessed by comparing species richness of each PA against richness estimated by a null model. Federal and state PAs are subdivided into two categories: Sustainable Use (SU) and Full Protection (FP). PAs assigned with (*) were considered as effective. See methods for further details.

**Names**	**Type**	**Category**	**All variables combined**		**Critical Average Exposure Temperature**		**Extreme Temperature Anomaly**		**Monthly Extreme Temperature**		**Extreme Rainfall Anomaly**		**Effective?**	
			**2050**	**2070**	**2050**	**2070**	**2050**	**2070**	**2050**	**2070**	**2050**	**2070**	**2050**	**2070**
Área de Proteção Ambiental Meandros do Rio Araguaia	Federal	SU	5	85	1	34	0	0	1	8	1	1	No	No
Estação Ecológica da Terra do Meio	Federal	FP	28	132	16	79	1	3	6	24	3	4	No	No
Parque Nacional de Anavilhanas	Federal	FP	41	155*	31	102*	1	3	13	24	1	1	No	Yes
Estação Ecológica de Iquê	Federal	FP	13	21	0	1	0	1	1	11	2	2	No	No
Estação Ecológica de Jutaí-Solimões	Federal	FP	38	111	32	96	0	0	2	19	0	0	No	No
Estação Ecológica Niquía	Federal	FP	27	135	17	82	0	0	5	11	1	1	No	No
Estação Ecológica do Jari	Federal	FP	28	132	20	81	0	0	11	18	2	2	No	No
Estação Ecológica Juami-Japurá	Federal	FP	30	87	26	86	0	0	2	11	0	0	No	No
Estação Ecológica Serra Geral do Tocantins	Federal	FP	3	77	0	9	0	0	0	7	0	0	No	No
Floresta Nacional de Altamira	Federal	SU	26	121	14	68	1	3	2	19	3	3	No	No
Floresta Nacional de Anauá	Federal	SU	33	136	24	85	0	0	11	18	1	1	No	No
Floresta Nacional de Balata-Tufari	Federal	SU	40	141	23	88	0	1	5	32	2	2	No	No
Floresta Nacional de Carajás	Federal	SU	10	65	6	58	0	1	2	12	2	2	No	No
Floresta Nacional de Caxiuana	Federal	SU	31	132	23	79	0	1	10	25	3	4*	No	Yes
Floresta Nacional de Humaitá	Federal	SU	36	134	17	80	1	4	6	28	1	1	No	No
Floresta Nacional de Itaituba II	Federal	SU	40	138	22	85	3*	7*	7	31	2	2	Yes	Yes
Floresta Nacional de Jacundá	Federal	SU	33	129	16	75	1	2	4	26	1	1	No	No
Floresta Nacional de Pau-Rosa	Federal	SU	45	146	26	93	3*	11*	13	35	2	2	Yes	Yes
Floresta Nacional de Roraima	Federal	SU	26	136	16	82	0	0	6	11	2	2	No	No
Floresta Nacional de Saracá-Taquera	Federal	SU	37	138	28	87	0	3	15*	24	1	1	Yes	No
Floresta Nacional de Tefé	Federal	SU	48	152	33	98	0	5	12	37	1	1	No	No
Floresta Nacional do Amapá	Federal	SU	32	99	24	84	0	0	16*	18	3	3	Yes	No
Floresta Nacional do Amazonas	Federal	SU	29	141	19	86	0	0	3	11	1	1	No	No
Floresta Nacional do Jatuarana	Federal	SU	31	126	11	72	1	4	5	21	2	2	No	No
Floresta Nacional do Macauã	Federal	US	22	92	14	88	0	0	7	38	2	2	No	No

Names	Type	Category	All variables combined		Critical Average Exposure Temperature		Extreme Temperature Anomaly		Monthly Extreme Temperature		Extreme Rainfall Anomaly		Effective?	
			**2050**	**2070**	**2050**	**2070**	**2050**	**2070**	**2050**	**2070**	**2050**	**2070**	**2050**	**2070**
Floresta Nacional do Purus	Federal	SU	40	141	25	90	0	0	6	34	2	2	No	No
Floresta Nacional do Tapajós	Federal	SU	47	149	28	96	3*	9*	13	38	3	3	Yes	Yes
Floresta Nacional do Tapirapé-Aquiri	Federal	US	7	60	3	55	0	1	2	10	3	3	No	No
Floresta Nacional Mapiá - Inauini	Federal	SU	24	142	17	88	0	0	6	35	2	2	No	No
Parque Nacional Da Amazônia	Federal	FP	41	141	23	88	1	8*	10	32	2	2	No	Yes
Parque Nacional Da Serra do Divisor	Federal	FP	51*	158*	39*	106*	0	0	13*	46*	2	2	Yes	Yes
Parque Nacional Da Serra do Pardo	Federal	FP	10	114	4	62	0	2	2	16	3	3	No	No
Parque Nacional Das Nascentes do Rio Parnaiba	Federal	FP	5	81	0	12	0	0	0	9	0	0	No	No
Parque Nacional de Pacaás Novos	Federal	FP	23	116	3	32	0	0	3	25	2	2	No	No
Parque Nacional do Araguaia	Federal	FP	7	90	2	39	0	0	1	10	1	1	No	No
Parque Nacional do Cabo Orange	Federal	FP	33	85	24	82	0	0	18*	23	3	3	Yes	No
Parque Nacional do Jaú	Federal	FP	36	146	28	93	0	2	5	18	1	1	No	No
Parque Nacional do Pico Da Neblina	Federal	FP	32	146	23	91	0	0	3	13	0	0	No	No
Parque Nacional do Viruá	Federal	FP	32	141	23	87	0	0	11	17	1	1	No	No
Parque Nacional Montanhas do Tumucumaque	Federal	FP	28	95	20	87	0	0	18*	25	3	3	Yes	No
Parque Nacional Serra da Cutia	Federal	FP	27	117	10	65	0	0	2	25	2	2	No	No
Parque Nacional Serra da Mocidade	Federal	FP	27	137	17	83	0	0	5	11	1	1	No	No
Reserva Biológica do Guaporé	Federal	FP	24	113	5	59	0	0	3	25	3	3	No	No
Reserva Biológica do Gurupi	Federal	FP	16	108	11	57	0	0	2	13	2	2	No	No
Reserva Biológica do Jaru	Federal	FP	26	115	3	64	1	3	4	20	1	1	No	No
Reserva Biológica do Lago Piratuba	Federal	FP	27	126	20	75	0	0	14*	15	2	2	Yes	No
Reserva Biológica do Rio Trombetas	Federal	FP	37	138	28	87	0	2	15	24	1	1	No	No
Reserva Biológica do Uatumã	Federal	FP	33	137	24	86	0	1	11	20	1	1	No	No
Reserva Biológica Nascentes da Serra do Cachimbo	Federal	FP	14	103	1	18	1	1	1	10	2	2	No	No
Reserva Extrativista Chico Mendes	Federal	SU	52*	152	36	100	0	0	14	48*	2	2	Yes	Yes
Reserva Extrativista do Cazumbá-Iracema	Federal	SU	25	96	15	88	0	0	8	39	2	2	No	No
Reserva Extrativista do Médio Juruá	Federal	SU	50*	156*	36*	103*	0	2	14*	42*	2	2	Yes	Yes
Reserva Extrativista do Rio Jutaí	Federal	SU	38	108	31	93	0	0	9	22	0	0	No	No
Reserva Extrativista do Lago do Capanã Grande	Federal	SU	37	136	20	82	1	4	5	27	1	1	No	No
Reserva Extrativista Riozinho da Liberdade	Federal	SU	48*	117	34*	102*	0	0	13*	40	2	2	Yes	Yes
Reserva Extrativista Riozinho do Anfrísio	Federal	SU	31	128	19	75	2*	4	6	24	2	3	Yes	No
Reserva Extrativista Tapajós-Arapiuns	Federal	SU	48	152	29	99	2*	8*	13	38	3	4*	Yes	Yes
Reserva Extrativista Verde Para Sempre	Federal	SU	42	149	30	95	1	3	13	35	3	4	No	No

Names	Type	Category	All variables combined		Critical Average Exposure Temperature		Extreme Temperature Anomaly		Monthly Extreme Temperature		Extreme Rainfall Anomaly		Effective?	
			2050	2070	2050	2070	2050	2070	2050	2070	2050	2070	2050	2070
Parque Nacional do Rio Novo	Federal	FP	17	108	6	55	1	1	0	12	1	1	No	No
Floresta Nacional do Trairão	Federal	SU	29	125	17	72	2*	4	4	20	2	2	Yes	No
Floresta Nacional do Jamanxim	Federal	SU	23	117	11	63	1	3	1	16	1	1	No	No
Parque Nacional do Jamanxim	Federal	FP	25	120	13	67	2*	4	3	17	2	2	Yes	No
Área de Proteção Ambiental do Tapajós	Federal	SU	36	134	17	80	2*	6	5	28	2	2	Yes	No
Floresta Nacional do Crepori	Federal	SU	22	116	11	62	1	2	0	14	1	1	No	No
Floresta Nacional do Amana	Federal	SU	38	133	20	80	2*	6*	7	28	2	2	Yes	Yes
Parque Nacional da Chapada Das Mesas	Federal	FP	11	93	6	43	0	0	0	12	1	1	No	No
Reserva Extrativista de Cururupu	Federal	SU	14	96	9	51	0	0	0	1	2	2	No	No
Parque Nacional do Juruena	Federal	FP	36*	132	15*	78*	3*	5*	5	28*	3	3	Yes	Yes
Reserva Extrativista Rio Unini	Federal	SU	32	142	24	90	0	2	4	13	1	1	No	No
Parque Nacional Dos Campos Amazônicos	Federal	FP	35	130	14	76	2*	6*	8	26	2	2	Yes	Yes
Reserva Extrativista Gurupá-Melgaço	Federal	SU	28	131	20	79	0	0	9	22	2	2	No	No
Reserva Extrativista do Alto Juruá	Federal	SU	51*	155	37*	104*	0	0	15*	46*	2	2	Yes	Yes
Reserva Extrativista do Rio Cajari	Federal	SU	34	137	25	85	0	0	14	24	3	4*	No	Yes
Reserva Extrativista do Médio Purus	Federal	SU	41	143	25	90	0	0	7	34	2	2	No	No
Floresta Nacional do Iquiri	Federal	SU	41	141	25	88	0	0	5	34	2	2	No	No
Parque Nacional Nascentes do Lago Jari	Federal	FP	42	142	26	88	2*	3	6	29	1	1	Yes	No
Reserva Extrativista Ituxi	Federal	SU	37	137	21	84	0	0	3	30	2	2	No	No
Parque Nacional Mapinguari	Federal	FP	43*	145*	26*	90*	1	2	7	37*	2	2	Yes	Yes
Estação Ecológica Alto Maués	Federal	FP	34	129	15	76	1	5	7	25	2	2	No	No
Parque Estadual Serra do Aracá	State	FP	25	138	15	83	0	0	2	8	1	1	No	No
Parque Estadual Igarapés do Juruena	State	FP	28	123	9	69	1	3	4	21	3	3	No	No
Parque Estadual de Guajará-Mirim	State	FP	33	107	23	88	0	0	15*	23	3	3	Yes	No
Parque Estadual do Araguaia	State	FP	37	146	27	94	0	0	16*	24	2	2	Yes	No
Estação Ecológica do Grão Pará	State	FP	5	78	0	32	0	0	0	7	0	0	No	No
Parque Estadual do Matupiri	State	FP	11	100	0	7	0	0	1	20	3	3	No	No
Parque Estadual Sucunduri	State	FP	42	144	23	90	2	4	5	31	2	2	No	No
Parque Estadual de Corumbiara	State	FP	5	86	1	34	0	0	1	8	1	1	No	No
Parque Estadual Chandless	State	FP	30	120	9	67	1	4*	4	24	3*	3	Yes	Yes
Parque Estadual do Jalapão	State	FP	28	120	12	67	1	1	4	26	1	1	No	No
Reserva Biológica de Maicuru	State	FP	26	96	18	92	0	0	9	42*	2	2	No	Yes
Floresta Estadual Sucunduri	State	SU	28	119	7	65	0	3	4	21	3	3	No	No

Names	Type	Category	All variables combined		Critical Average Exposure Temperature		Extreme Temperature Anomaly		Monthly Extreme Temperature		Extreme Rainfall Anomaly		Effective?	
			**2050**	**2070**	**2050**	**2070**	**2050**	**2070**	**2050**	**2070**	**2050**	**2070**	**2050**	**2070**
Floresta Estadual Maúes	State	SU	41	140	23	87	1	7*	9	31	2	2	No	Yes
Área de Proteção Ambiental do Rio Preto	State	SU	3	79	0	0	0	0	0	9	0	0	No	No
Área de Proteção Ambiental do Arquipélogo do Marajó	State	SU	46	154*	34	101*	0	0	21*	37	4*	4*	Yes	Yes
Área de Proteção Ambiental do Lago de Tucurui	State	SU	26	125	18	72	0	1	4	22	3	3	No	No
Floresta Estadual de Faro	State	SU	37	137	28	86	0	2	15	24	1	1	No	No
Floresta Estadual de Iriri	State	SU	24	115	13	63	1	3	3	18	3	3	No	No
Floresta Estadual do Trombetas	State	SU	38	143	28	92	0	2	17*	25	2	2	Yes	No
Floresta Estadual do Paru	State	SU	36	142	27	90	0	1	15	24	3	3	No	No
Área de Proteção Ambiental Triunfo do Xingu	State	SU	12	118	4	65	0	2	2	17	3	3	No	No
Área de Proteção Ambiental Ilha do Bananal/Cantão	State	SU	10	95	5	44	0	0	1	11	2	2	No	No
Reserva Extrativista do Rio Gregório	State	SU	46	149	32	98	0	0	10	37	2	1	No	No
Reserva de Desenvolvimento Sustentável do Juma	State	SU	38	135	18	81	4*	7*	8	28	2	2	Yes	Yes
Floresta Estadual de Tapauá	State	SU	38	139	23	86	0	1	4	28	1	1	No	No
Reserva de Desenvolvimento Sustentável Igapó-Açu	State	SU	37	136	20	83	1	1	3	25	1	1	No	No
Reserva Extrativista Canutama	State	SU	39	142	24	89	0	0	7	32	2	2	No	No
Reserva de Desenvolvimento Sustentável do Matupiri	State	SU	37	135	20	82	1	1	4	25	1	1	No	No
Área de Proteção Ambiental das Reentrôncias Maranhenses	State	SU	15	101	10	53	0	0	1	3	2	2	No	No
Área de Proteção Ambiental da Baixada Maranhense	State	SU	14	100	9	53	0	0	1	11	2	2	No	No
Área de Proteção Ambiental de Upaon-Açu / Miritiba / Alto Preguiças	State	SU	15	97	10	52	0	0	1	3	2	2	No	No
Área de Proteção Ambiental - Baixo Rio Branco	State	SU	36	147	26	95	0	1	12	20	1	1	No	No
Reserva de Desenvolvimento Sustentavel do Rio Madeira	State	SU	43	141	23	88	4*	7*	11	33	2	2	Yes	Yes
Reserva de Desenvolvimento Sustentável do Rio Iratapuru	State	SU	30	135	23	84	0	0	14	20	3	3	No	No
Área de Proteção Ambiental Das Cabeceiras do Rio Cuiabá	State	SU	3	22	0	14	0	0	0	5	1	1	No	No
Área de Proteção Ambiental Da Chapada Dos Guimarães	State	SU	2	80	0	14	0	0	0	8	1	1	No	No
Reserva Extrativista Rio Pacás Novos	State	SU	27	117	10	65	0	0	2	25	2	2	No	No
Reserva Extrativista Rio Preto-Jacundá	State	SU	29	123	11	70	1	4	5	23	1	1	No	No
Floresta Estadual do Amapá	State	SU	36	141	25	90	0	0	18*	25	3	3	Yes	No
Reserva de Desenvolvimento Sustentável Amanã	State	SU	50	159	36	105	1	4	8	32	1	1	No	No
Reserva de Desenvolvimento Sustentável Cujubim	State	SU	45	115	34	100	0	0	12	35	1	1	No	No
Reserva de Desenvolvimento Sustentável Mamirauá	State	SU	57*	168*	44*	114*	1	5	15	40	0	0	Yes	Yes
Reserva de Desenvolvimento Sustentável Piagaãu Purus	State	SU	46	149	30	95	2*	4	10	33	2	2	Yes	No
Reserva de Desenvolvimento Sustentável Rio Amapá	State	SU	38	137	21	83	1	2	3	26	1	1	No	No
Reserva de Desenvolvimento Sustentável Uacari	State	SU	52*	159*	38*	106*	0	2	15	44*	2	2	Yes	Yes

Names	Type	Category	All variables combined		Critical Average Exposure Temperature		Extreme Temperature Anomaly		Monthly Extreme Temperature		Extreme Rainfall Anomaly		Effective?	
			**2050**	**2070**	**2050**	**2070**	**2050**	**2070**	**2050**	**2070**	**2050**	**2070**	**2050**	**2070**
Reserva de Desenvolvimento Sustentável do Uatumã	State	SU	39	144	29	92	0	3	14*	25	1	1	Yes	No
Reserva Extrativista Catuá-Ipixuna	State	SU	46	149	32	96	1	5*	10	32	2	1	No	Yes
Área de Proteção Ambiental de Presidente Figueiredo - Caverna do Moroaga	State	SU	34	139	25	87	0	1	11	21	1	1	No	No
Área de Proteção Ambiental Margem Direita do Rio Negro- Setor Paduari-Solimıes	State	SU	34	144	25	91	0	2	7	18	1	1	No	No
Área de Proteção Ambiental Margem Esquerda do Rio Negro-Setor Aturiá-Apuauzinho	State	SU	44	159	34	106	1	3	15	27	1	1	No	No
Área de Proteção Ambiental Nhamundá	State	SU	49*	156*	30	103	0	7*	17*	36	2	2	Yes	Yes
Floresta Estadual Apuí	State	SU	26	117	7	63	0	2	3	19	3	3	No	No
Floresta Estadual Aripuanã	State	SU	28	123	9	69	0	3	4	20	2	2	No	No
**Total**													36	27
